# Children’s everyday exposure to food marketing: an objective analysis using wearable cameras

**DOI:** 10.1186/s12966-017-0570-3

**Published:** 2017-10-08

**Authors:** L. N. Signal, J. Stanley, M. Smith, M. B. Barr, T. J. Chambers, J. Zhou, A. Duane, C. Gurrin, A. F. Smeaton, C. McKerchar, A. L. Pearson, J. Hoek, G. L. S. Jenkin, C. Ni Mhurchu

**Affiliations:** 1 Department of Public Health, Health Promotion & Policy Research Unit, University of Otago, PO Box 7343, Wellington South, Wellington, 6242 New Zealand; 2 Insight Centre for Data Analytics, Dublin City University, Belfield, Dublin, Ireland; 3 Department of Geography, Environment and Spatial Sciences, Michigan State University, 673 Auditorium Rd, East Lansing, MI 48825 USA; 4 Department of Marketing, University of Otago, Level 4, Business School, Clyde St, North Dunedin, Dunedin, 9016 New Zealand; 5 National Institute for Health Innovation, University of Auckland, 261 Morrin Road, Glen Innes, Auckland, 1072 New Zealand

**Keywords:** Food marketing, Childhood obesity, Obesogenic environments, Wearable cameras

## Abstract

**Background:**

Over the past three decades the global prevalence of childhood overweight and obesity has increased by 47%. Marketing of energy-dense nutrient-poor foods and beverages contributes to this worldwide increase. Previous research on food marketing to children largely uses self-report, reporting by parents, or third-party observation of children’s environments, with the focus mostly on single settings and/or media. This paper reports on innovative research, Kids’Cam, in which children wore cameras to examine the frequency and nature of everyday exposure to food marketing across multiple media and settings.

**Methods:**

Kids’Cam was a cross-sectional study of 168 children (mean age 12.6 years, SD = 0.5) in Wellington, New Zealand. Each child wore a wearable camera on four consecutive days, capturing images automatically every seven seconds. Images were manually coded as either recommended (core) or not recommended (non-core) to be marketed to children by setting, marketing medium, and product category. Images in convenience stores and supermarkets were excluded as marketing examples were considered too numerous to count.

**Results:**

On average, children were exposed to non-core food marketing 27.3 times a day (95% CI 24.8, 30.1) across all settings. This was more than twice their average exposure to core food marketing (12.3 per day, 95% CI 8.7, 17.4). Most non-core exposures occurred at home (33%), in public spaces (30%) and at school (19%). Food packaging was the predominant marketing medium (74% and 64% for core and non-core foods) followed by signs (21% and 28% for core and non-core). Sugary drinks, fast food, confectionary and snack foods were the most commonly encountered non-core foods marketed. Rates were calculated using Poisson regression.

**Conclusions:**

Children in this study were frequently exposed, across multiple settings, to marketing of non-core foods not recommended to be marketed to children. The study provides further evidence of the need for urgent action to reduce children’s exposure to marketing of unhealthy foods, and suggests the settings and media in which to act. Such action is necessary if the Commission on Ending Childhood Obesity’s vision is to be achieved.

**Electronic supplementary material:**

The online version of this article (doi:10.1186/s12966-017-0570-3) contains supplementary material, which is available to authorized users.

## Background

Over the past three decades the global prevalence of childhood overweight and obesity has increased by 47% [1]. Excess adiposity during childhood and adolescence is associated with an increased risk of many serious health conditions and has lifetime consequences for children’s health, well-being, and productivity [2–4].

Marketing of energy-dense nutrient-poor (EDNP) foods and beverages contributes to the worldwide increase in childhood obesity [5] by encouraging the repeat purchase and consumption of foods that do not meet nutritional guidelines [6–8]. Internationally, it is estimated that 60% to 90% of food marketing to children is for pre-sugared breakfast cereals, soft drinks, savoury snacks, confectionery and fast foods [8]. The World Health Organization (WHO) Commission on Ending Childhood Obesity (ECHO) recommends reducing children’s exposure to, and the power of, marketing of unhealthy foods [5]. ECHO states that “settings where children and adolescents gather (such as schools and sports facilities or events) and the screen-based offerings they watch or participate in, should be free of marketing of unhealthy food and sugar-sweetened beverages” [5, p.18]. According to the WHO Regional Office for Europe Nutrient Profiling Model [9], foods not recommended to be marketed to children include confectionery, sweet snack food, ice-cream, iced confectionery and sugar-sweetened and artificially-sweetened beverages. In New Zealand, the industry self-regulating Children’s Code for Advertising Food states that “food advertisements should not undermine the food and nutrition policies of Government, the Ministry of Health Food and Nutrition Guidelines nor the health and well-being of children” ([10], p.21.)

Previous studies quantifying children’s exposure to food and beverage marketing have concluded that promotions encouraging the consumption of EDNP products are ubiquitous in children’s environments [6, 11–15]. Yet, despite this important work, little is known about children’s actual daily exposure to food marketing. This knowledge gap exists because previous research has largely used self-report, reporting by parents, or third-party observation of children’s environments. Further, it often focuses on single settings [16–18] (outdoors) and/or media (television) [11, 14, 19, 20]. This paper reports on innovative research, Kids’Cam, which used wearable cameras to examine the frequency and nature of New Zealand (NZ) children’s everyday exposure to food and non-alcoholic beverage marketing (hereafter food marketing) across multiple media and settings [21]. Marketing exposure was examined by socioeconomic status and ethnicity (including whether the magnitude of any ethnic differences varied with socioeconomic status), as childhood obesity is strongly patterned by these factors [21].

## Methods

### Study design

Kids’Cam was a cross-sectional study of 168 Year 8 children (typical age range 11 to 13 years) in the Wellington region of NZ. Children were asked to wear a camera around their neck from when they got up in the morning until going to bed for four consecutive days (Thursday to Sunday, to capture both weekday and weekend exposures). They were advised to remove the camera in situations where privacy could be expected (e.g. toilet or shower facilities), if they felt uncomfortable, when swimming or playing vigorous sport, or if requested. The camera automatically captured a 136° image of the front-facing scene approximately every seven seconds. Data were collected over a 12-month period from July 2014 to June 2015 to allow for seasonal variations. Full details of the study methods (including sample size calculations) are published elsewhere [22]. The study protocol is available at https://diet.auckland.ac.nz/content/kidscam

### Sampling and recruitment

Sampling and recruitment were conducted in two stages, first at school level and then child level. The number of Year 8 children enrolled across all schools in the Wellington region was collated using aggregate school enrolment data from the Ministry of Education, and schools were sampled with probability-proportional-to-size stratified random sampling by school decile^1^ (low decile = 1–3, medium decile = 4–7, high decile = 8–10) and student ethnicity Māori (indigenous population), Pacific (mostly second generation migrants from Pacific Islands), and NZ European (NZE). This sampling strategy facilitated comparisons of marketing exposure by socioeconomic status and ethnicity, and gave a total of nine sampling strata. Randomly selected schools were invited to participate.

In consenting schools, a maximum of 20 Year 8 children were randomly selected from the class list, stratified by ethnicity, using R 3.2.4 (R Institute, Vienna). The school principal or lead teacher reviewed the list of students to identify children who did not meet the study criteria (*n* = 5 over the study period). The first 15 eligible children were invited to participate, and the first six children on the list who returned signed consent forms (including parental consent) were selected to participate. The number of children invited exceeded the number of participants required in order to achieve recruitment of four to six children per school (as per the sampling strategy), and reduce the burden on the schools from multiple rounds of invitation.

### Data collection and management

Written consent was gained from children and their parents and basic demographic data were collected via parental questionnaire. A briefing session was held with participating children the day before the cameras were first worn to explain the project. Following data collection, cameras were collected and images downloaded, with children given the opportunity to review and delete any photos before the researchers viewed them. At this review, height and weight were measured to determine age- and gender-specific BMI, using the extended international body mass index cut-offs [22]. Approved images were downloaded to a password-protected server, saved in secure cloud storage, and backed up to a password-protected external hard drive. Approximately 1.3 million images were recorded that could be coded for the presence of food marketing.

### Coding of image data

Image coding was performed using a coding protocol to guide content analysis [23]. Customised software enabled manual coding of each image. Marketing was defined as “any form of commercial communication or message that is designed to, or has the effect of, increasing recognition, appeal and/or consumption of particular products and services” ([24], p.9). A three-tiered framework was used to code each relevant image for setting, marketing medium and food product category, based on the WHO food marketing framework [9]. Key settings codes were home, school, food venues, recreation venues and other public spaces. Key marketing media codes were product packaging, signs, in-store marketing, print media, screen and merchandise.

MB, TC and four other health science students undertook the coding. A half day training workshop was held with all coders and coders were then given access to the dataset for a number of days to become familiar with it. Once coders felt comfortable, reliability testing was conducted, with each coder achieving 90% concurrence with model answers on a test dataset of 115 images before coding commenced. Coders were supervised by MS, MB and TC to ensure consistency. Uncertain codes were noted as such and checked by MB or TC.

All foods were classified as either recommended (core) or not recommended (non-core) to be marketed to children based on the WHO Regional Office for Europe Nutrient Profiling Model [9], with some modifications (e.g. a ‘fast food’ category was added which included all commercially prepared food products sold at quick service restaurants). All fast food was classified as not recommended to be marketed to children as it is typically high in saturated fat and sodium and low in fiber [25]. Marketing in convenience stores and supermarkets was too extensive to code individually and was therefore excluded from this analysis. Codes were only assigned to an image where 50% or more of a brand name or logo could be clearly seen by the coder. Individual images could be coded for multiple marketing media and product categories.

Further processing of the coded data included determining the number of marketing exposures for each unique exposure code (defined as the combination of setting, medium and product type for that code). A marketing exposure was defined as starting on the first instance of an image with a particular setting/medium/product code; subsequent images were counted as part of the same exposure. An exposure was considered to have ended when 30 s had elapsed since the last recorded code of that setting/medium/product code (defined using the image timestamps). Any subsequent code for that same combination after this 30 s limit was counted as the start of a new exposure sequence.

The number of exposures was summed for each unique exposure code by child; aggregate counts were determined for each child to estimate total exposures to core and non-core foods, and exposure by setting, medium, and product type. Cleaning and aggregation of coded data was completed in R version 3.2.3 (R Institute, Vienna).

### Data analysis

All statistical analysis was conducted in Stata 12 (StataCorp, College Station, TX, USA). Data analysis for study outcomes accounted for the complex sampling by using inverse sampling weights to account for over- and under-sampling of groups by ethnicity and school decile relative to their share of the Year 8 population in the Wellington region, and inferential statistics incorporated elements to handle sample stratification and clustering of children within schools (95% confidence intervals, *p*-values) [26] using Stata’s svy prefix commands and associated weighting options.

Descriptive analysis of the overall cohort was undertaken to describe children by ethnicity, school decile group, age, gender, individual deprivation (NZiDep) [27] and BMI status. Schools participating in the study were described by sub-region within the greater Wellington area and school decile group.

Descriptive analysis of rates of core and non-core food marketing exposures for each child was undertaken by taking the total number of exposures (by core and non-core foods) and dividing by the total number of photos for that child, with this number subsequently re-scaled as an exposure rate for a ten hour day. These were summarised within the major sampling groups (ethnicity and school decile stratum) as median and interquartile ranges of the daily rates, weighted for the sampling design.

Subsequent analysis of rates of marketing exposures used Poisson regression methods, as appropriate for count-based numerator data, analysed separately for core and non-core food marketing exposures. Rates and rate ratios were presented with 95% confidence intervals (95% CI). Results were reported as rates per day of photos (i.e. per 10 h of photographs). Each photo was specified as contributing seven seconds of exposure time (seven seconds being the median interval between images) for the Poisson regression.

Rates of core and non-core exposures per day were analysed using Poisson regression models. Separate models were constructed for core and non-core food exposures. For each, an initial model looked at differences by ethnicity, adjusted for child gender and age (treated as a linear covariate); a second model added school decile group (area level socioeconomic position) to this first model. A third model examined our primary research question of whether ethnic group differences in overall rates of marketing exposures differed across school decile group, by including interaction terms between these two variables. *P*-values are reported for hypothesis tests of these interaction terms and fully stratified results are presented when these hypothesis tests were significant. These results are presented in the additional files as rates within each ethnicity/school decile stratum, and as rate ratios comparing exposure rates between ethnic groups, as calculated separately within each school decile stratum.

## Results

### Participating schools and children

**Fig. 1:**
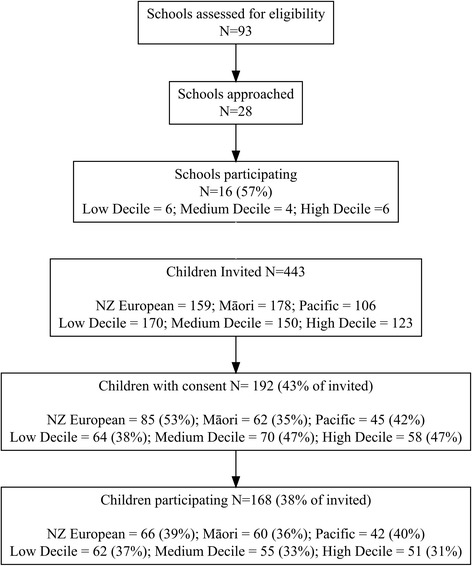
Sampling and recruitment flow diagrams for schools and children, by ethnicity and school decile stratum

Sampling and recruitment of schools and children are summarised in Fig. 1. All 93 schools with Year 8 students in the Wellington region were eligible to be sampled. Twenty-eight schools were approached across the nine sampling strata and 16 consented to participate (57%). Of the 443 children invited to participate, 192 gave consent (43%) and 168 participated (38%). Sociodemographic information for participating children is presented in Table 1. Most children were 12 years old (75%: mean = 12.6 years, SD = 0.5) with approximately equal numbers of girls and boys (52.7% female). Just over half the children were of normal weight or underweight (57.5%); with the remainder overweight or obese (42.5%). The lower part of Table 1 shows location and school decile for participating schools. The number of children in each sampling stratum is reported in Additional file 1, along with a summary of the number of photos available for analysis within each stratum.

**Table 1:** Sociodemographic and other characteristics of Kids’Cam participants and schools

Sociodemographic variable	Group	N (%)
Child participants (total *n* = 168)		
Ethnicity	NZ European	66 (39.3)
	Māori	60 (35.7)
	Pacific	42 (25.0)
School decile	Low (1–3)	62 (36.9)
	Medium (4–7)	55 (32.7)
	High (8–10)	51 (30.2)
Age (years)*	11	13 (8.0)
	12	122 (75.3)
	13	26 (16.1)
	14	1 (0.6)
Gender	Female	88 (52.7)
	Male	80 (47.3)
NZiDep *	1	52 (32.1)
	2	33 (20.4)
	3	25 (15.4)
	4	26 (16.1)
	5	26 (16.1)
BMI**	Underweight	9 (5.4)
	Healthy	87 (52.1)
	Overweight	46 (27.5)
	Obese	25 (15.0)
School details (*n* = 16)		
Location		
	Wellington	6 (37.5)
	Porirua	6 (37.5)
	Hutt Valley	4 (25.0)
School decile***		
	Low (1–3)	7 (43.8)
	Medium (4–7)	3 (18.8)
	High (8–10)	6 (37.5)

* Age and NZiDep missing for 6 participants (questionnaire not completed)

** BMI missing for 1 participant as child declined to be measured

*** Some schools were sampled multiple times for a particular ethnicity/school decile stratum in accordance with sampling probability-proportional-to-size

### Rates of marketing exposures

Rates of marketing exposures per day for core and non-core foods are presented in Table 2. The mean rate for core food was 12.3 marketing exposures per day; for non-core foods, the mean rate was 27.3 marketing exposures per day, more than twice that for core foods. Additional file 2 reports the median and interquartile range of daily exposure to core and non-core food marketing: the interquartile range spread from 15 to 34 non-core exposures per day.

**Table 2:** Mean rate of core and non-core food marketing exposures (per day, with 95% CI, from Poisson regression) for total exposures (across all settings/media) and by setting, medium, and product category (with percentage share of all exposures by setting/medium/product category)

	Core Foods		Non-core Foods	
Total/Setting/Medium/Product category	Rate per day* (95% CI)	% of total	Rate per day* (95% CI)	% of total
Total (any setting/ marketing medium)	12.3 (8.7, 17.4)	100	27.3 (24.8, 30.1)	100
Setting**				
Home	5.5 (4.6, 6.6)	44.9	8.9 (7.9, 10.1)	32.8
School	5.3 (2.9, 9.5)	42.9	5.3 (4.2, 6.8)	19.5
Food venues***	0.2 (0.1, 0.4)	1.7	2.7 (1.5, 4.7)	9.7
Recreation venues****	0.4 (0.3, 0.7)	3.5	2.1 (1.1, 3.8)	7.6
Other public spaces*****	0.9 (0.5, 1.5)	7.0	8.3 (6.0, 11.4)	30.4
Marketing medium				
Product packaging	9.1 (7.2, 11.4)	73.5	17.4 (15.7, 19.4)	63.9
Sign	2.6 (1.0, 6.8)	21.2	7.6 (5.3, 10.9)	27.9
Instore marketing	0.1 (0.0, 0.2)	0.6	1.0 (0.7, 1.4)	3.6
Print media	0.0 (0.0, 0.1)	0.2	0.6 (0.2, 1.8)	2.2
Screen	0.1 (0.0, 0.2)	0.5	0.2 (0.1, 0.4)	0.6
Merchandise	0.5 (0.2, 1.2)	3.9	0.5 (0.2, 1.2)	1.9
Product category				
Core	12.3 (8.7, 17.4)	100		
Sugary drinks			9.1 (8.3, 10.0)	33.4
Fast food			6.0 (4.7, 7.6)	22.1
Confectionery			3.0 (2.3, 4.0)	11.1
Snack foods			2.9 (2.4, 3.5)	10.5
Ice cream			1.9 (1.3, 2.7)	7.0
Diet soft drinks			1.4 (0.9, 1.9)	4.9
Cookies/cakes/pastries			1.3 (0.9, 2.0)	4.8
Milk product (unhealthy)			0.8 (0.4, 1.3)	2.8
Cereal (unhealthy)			0.7 (0.4, 1.1)	2.5
Other			0.2 (0.1, 0.4)	0.9

* Rate of marketing exposures per day (calculated as rate per 10 h of photographs)

** Details for aggregated settings are presented in Additional file 2

*** Includes fast food indoor, full service restaurant, and fresh food market

**** Includes sport, outdoor recreation, and community venue

***** Includes street, shop front, shopping mall, private transport, public transport facility, onboard public transport, and other retail

**Fig. 2:**
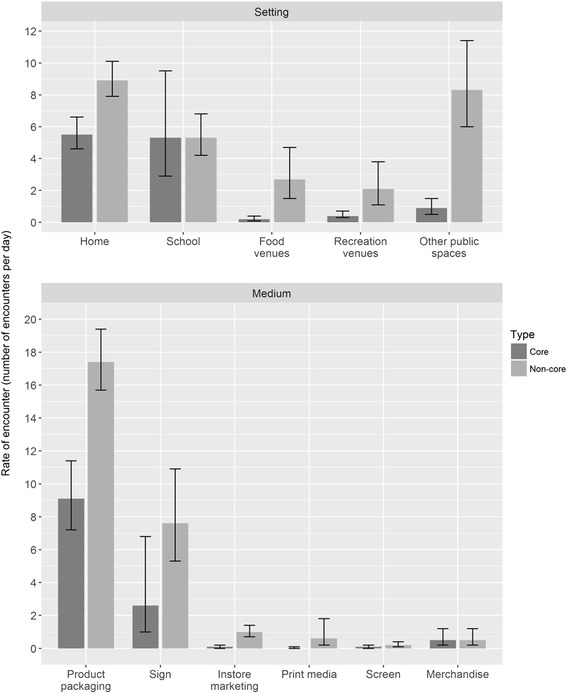
Mean rate (and 95% CI) of core and non-core food marketing exposures per day (10 h of photographs) by setting (top panel) and medium (bottom panel)

Most core food marketing exposures occurred at home or school (5.5 and 5.3 exposures per day, making up 45% and 43% of all core exposures respectively) (see Table 2 and Fig. 2, top panel); for non-core food marketing exposures, the majority happened either at home (33% of all non-core exposures) or in public spaces other than food or recreation venues (30% of all non-core exposures). One-fifth of non-core food marketing exposures occurred at school (19%). Additional file 3 gives further detail regarding the settings in which marketing exposures occurred: for example, most exposures in other public spaces were on the street or on shop fronts.

**Fig. 3:**
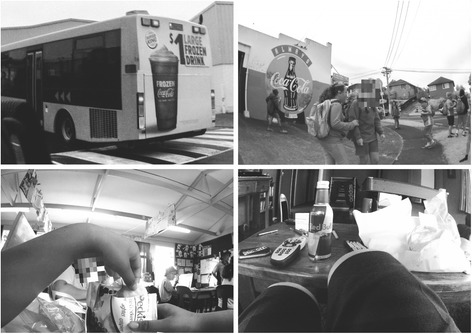
Sign for sugary drink in public space, sign for sugary drink in public place, product packaging for snack food at school, product packaging for sugary drink at home

The majority of marketing exposures were in the form of food packaging (see Table 2 and Fig. 2, bottom panel), at a mean rate of 9.1 exposures per day for core foods (74% of core exposures) and 17.4 exposures for non-core foods (64% of non-core exposures). The remaining marketing exposures were mostly signs (21% and 28% of core and non-core food marketing exposures, respectively) (see Fig. 3 for images of marketing).

### Types of non-core food product marketing exposures

Marketing exposure rates for specific non-core food product categories are presented in Table 2. The largest share was for sugary drinks (mean rate of 9.1 exposures per day, 33% of non-core exposures) followed by fast food (22% of non-core exposures), confectionery (11% of non-core exposures) and snack foods (10% of non-core exposures). Foods making up the remainder of non-core marketing exposures (24% of exposures) are listed in Table 2.

### Rates of marketing exposures by child ethnicity and school decile stratum

The mean exposure rates for core and non-core foods are presented in Additional file 4, stratified by ethnicity and school decile stratum. The rate of exposure for non-core foods was higher than for core foods in all strata.

Initial analysis for core foods compared exposure by ethnicity, adjusted for gender and age (Table 3, model 1). Māori children had non-significantly higher rates of exposure compared to NZE (RR = 1.55, 95% CI 0.68, 3.56); and Pacific children had similar rates of exposure to NZE (RR = 0.98, 95% CI 0.59, 1.61). Adding school decile group into the model did not appreciably change ethnic differences (Table 3, model 2). Compared to middle-decile children, children at higher decile schools had higher exposure to core foods (RR = 1.60, 95% CI 1.03, 2.48); while children at lower decile schools had non-significantly higher rates of such exposure (RR = 1.18; 95% CI 0.80, 1.73; reference is middle decile group). The third model incorporated a formal interaction test between ethnicity and school decile group, which was non-significant, suggesting that ethnic patterns were similar across school decile groups (F 4, 15 = 1.99; *p* = 0.1481).

**Table 3:** Rate ratios (with 95% confidence intervals) from Poisson regression models for core and non-core food exposures, from models accounting for ethnicity, gender, and age (model 1) and extended model including school decile group (model 2)

	Core Foods	Non-core Foods
Variable	Rate ratio (95% CI)	Rate ratio (95% CI)
MODEL 1. Ethnicity, Gender, Age		
Ethnicity		
NZE	1 (reference)	1 (reference)
Māori	1.55 (0.68, 3.56)	1.18 (0.90, 1.55)
Pacific	0.98 (0.59, 1.61)	0.99 (0.84, 1.16)
Gender		
Female	1 (reference)	1 (reference)
Male	0.84 (0.62, 1.15)	1.03 (0.83, 1.27)
Age (per year*)	1.28 (0.98, 1.69)	0.97 (0.78, 1.21)
MODEL 2. Model 1 + School decile group		
Ethnicity		
NZE	1 (reference)	1 (reference)
Māori	1.70 (0.78, 3.69)	1.23 (0.94, 1.62)
Pacific	1.15 (0.70, 1.88)	1.06 (0.91, 1.23)
Gender		
Female	1 (reference)	1 (reference)
Male	0.88 (0.61, 1.28)	1.03 (0.81, 1.30)
Age (per year*)	1.19 (0.90, 1.58)	0.97 (0.79, 1.21)
School decile group		
Low (1–3)	1.18 (0.80, 1.73)	0.90 (0.77, 1.06)
Middle (4–7)	1 (reference)	1 (reference)
High (8–10)	1.60 (1.03, 2.48)	1.05 (0.87, 1.27)

* Rate ratio for a one year difference in age

Analysis of ethnic differences in non-core exposures (adjusted for child gender and age; Table 3, right hand column, model 1) showed non-significantly higher rates of exposure to non-core foods for Māori children relative to NZE (RR = 1.18, 95% CI 0.90, 1.55) but not for Pacific children (RR = 0.99, 95% CI 0.84, 1.16). Differences in exposure by school decile group appeared minimal (Table 3) and adjustment of ethnic differences for school decile group did not appreciably change estimates from those in the initial model. A third model, incorporating interaction terms, suggested that ethnic differences in non-core exposures differed across the three school decile groups (F 4, 15 = 4.58, *p* = 0.013). These results are presented in Additional files 4 and 5. In brief, there was reasonably strong evidence for ethnic differences in the lowest school decile group (Māori RR = 1.20, 95% CI 0.97, 1.47; Pacific RR = 1.50, 95% CI 1.19, 1.89; both relative to NZE).

## Discussion

Children in this study were exposed to non-core food marketing, food not recommended to be marketed to children, 27.3 times a day on average across all settings, excluding convenience stores and supermarkets. Exposure to non-core food marketing was more than twice that of exposure to core food marketing (12.3 times a day). Most non-core exposures occurred at home, in public spaces and at school. Food packaging was the predominant marketing medium, followed by signs. Product packaging is commonly used to attract attention, provide information about product attributes and encourage purchase at point-of-sale [16]. Product packaging is particularly salient as children are the population group most vulnerable to such food marketing [20].

Children were most exposed to non-core marketing for sugary drinks, fast food, confectionary and snack foods, a finding consistent with previous research [11, 14, 28–31]. A notable exception is exposure to marketing for high-sugar, low-fibre breakfast cereals which comprised only 2.5% of all non-core marketing. Research in the UK and Australia found high rates of such marketing on television [11, 30].

Although televisions, smart phones, tablets and computers often appeared in the images, screen-based marketing is likely under-reported in the current study as content on screens was often not clear enough to meet coding criteria in the images. Research across 11 countries in 2010 reported five food advertisements per hour of television. A 2014 national survey of NZ children aged 6–14 found 88% watch television each day, 44% of whom watch more than an hour a day [32] thus potentially seeing five food advertisements daily on television alone, considerably more than the 0.2 exposures per day identified across all screen types in the current study. Food marketing on new media is also of concern (e.g. websites, social media and apps) and may have even greater impact than traditional media e.g. television [33]. NZ children engage with the internet frequently, with 66% accessing it daily [32].

Exposure to non-core food marketing was higher than for core foods in all school decile strata. Core exposures were more common in the high school decile groups; while for non-core exposures, there were no significant differences in exposure by these school decile groups. Similarly, while Māori children had higher exposure to both core and non-core marketing than NZE children, these results were not statistically significant in the adjusted models. The more complex model incorporating interaction terms suggested that ethnic group differences were somewhat varied across school decile group, with stronger evidence in the lowest school decile group for Pacific and Māori children.

To our knowledge, this is the first study to objectively measure children’s exposure to food marketing in their everyday environments across multiple settings and in multiple media. The use of automated wearable cameras enabled unprecedented access to children’s worlds, recording their exposures with food marketing as they occurred. This methodology overcomes many of the limitations inherent in using self-report or proxy report data [34]. Further, it comprehensively documented children’s actual exposure to marketing, with the important exceptions of marketing on screens, and in convenience stores and supermarkets. This is a major advantage of the Kids’Cam methodology: documenting actual exposure is challenging in third-party environmental observation studies, particularly in private contexts such as the home.

While this research provides some of the most robust data yet analysed on children’s exposure to food marketing, it does have limitations. First, the images do not determine if a child actually sees the marketing in the image. For example, the child could be looking away, although given the extent of food marketing in children’s environments they may still see marketing. Secondly, the decision to only code an image if 50% or more of a brand name or logo could be clearly seen is likely to underestimate the exposure to marketing, as does the exclusion of marketing in convenience stores and supermarkets, where marketing is likely to be extensive [35]. Further, the use of still photography may have missed some exposures. However, excluding screens, convenience stores and supermarkets, the ratio of more than two non-core food marketing exposures for every one core exposure is likely to be consistent, despite these limitations. The participation rate (*n* = 192 or 43% of invited children consenting to participate; with space for 168 participants, or 38% of the full invitation list participating) was reasonable for a study that required ongoing engagement by the children over several days. It remains possible that those children and families consenting to participate were systematically different from children who did not participate. Finally, while the sample size was determined prior to the study commencing, the number of participants was limited by the study budget and timeframe. This meant that some analyses (e.g. comparisons of exposure rates by ethnic group) might have had sub-optimal power to detect differences between groups, which is reflected in the relatively wide confidence intervals for these estimates. These specific estimates should be interpreted with caution.

Further real time research is needed on children’s exposure to marketing in convenience stores and supermarkets and on screens to complement this research. Further exploration of potential ethnic differences appears warranted, but will require a substantially larger sample size to improve statistical precision and power. Use of photo elicitation [36] with children who wore cameras would likely elicit valuable data on the meaning of food marketing and enable exploration of effective means for intervention from children’s perspective. Manual data coding was resource intensive, taking a total of 1440 person-hours. While this was an extensive undertaking, the richness of the resulting dataset made it worthwhile: the children collected 2553 h of image data from their perspective, giving insight into settings that would have been difficult to study as a participant observer. Ancillary studies also benefitted from this initial coding, as settings and other image characteristics were already available to researchers, which reduces processing times in these subsequent studies [37, 38]. Further, automated image recognition has the potential to aid analysis and reduce manual coding time requirements [39, 40]. The Kids’Cam method has the potential to validate other methods, e.g. surveys of school food policies, with in-depth analysis of the actual food environment [41]. Comparative research of children’s exposure to food marketing in other jurisdictions would further strengthen the global body of evidence.

This research suggests that children live in an obesogenic food marketing environment that promotes obesity as a normal response to their everyday environment [42]. Children are more than twice as likely to be exposed to non-core food marketing, not recommended to be marketed to children [9], than core food marketing, and to be exposed multiple times a day across various settings and via multiple media. All children, regardless of socio-economic position, were exposed to more non-core than core food marketing, and there appears to be some ethnic patterning.

Particularly concerning is the amount of exposure in school, an environment where children’s health is required to be protected under NZ law [43], and which the ECHO Commission states should be free of such marketing [5]. Exposure in public places is an arena for central and local governments globally. Given that over two-thirds of marketing is in the form of food packaging, consideration should be given to plain packaging in some specific cases (e.g. sugar sweetened beverages) as a highly effective intervention in this arena [44].

## Conclusions

The ECHO Commission is right to call for the reduction of children’s exposure to marketing of unhealthy foods [5]. This research provides further evidence of the need for action and suggests both settings and media in which to act. Urgent action is required if the vision of the Commission on Ending Childhood Obesity is to be achieved.

## Additional files

Additional file 1: Number of children and photos by ethnicity/school decile sampling stratum, and mean number of photos per child in each stratum. (DOCX 15 kb)

Additional file 2: Median and interquartile range of per-child rates of exposure per day to core and non-core items, by school. (DOCX 15 kb)

Additional file 3: Mean rate of core and non-core food marketing exposures (per day, with 95% CI, from Poisson regression) by setting with aggregated and detailed setting information (with percentage share of all exposures by setting). (DOCX 16 kb)

Additional file 4: Mean rate (and 95% CI) of core and non-core marketing exposures per day (10 h of photographs), by school decile stratum and ethnicity of child. (DOCX 98 kb)

Additional file 5: Rate ratios for differences in non-core food marketing exposures (from Poisson regression, with 95% CI) by interaction school decile group and ethnicity, adjusted for gender and age. (DOCX 15 kb)

## Footnotes

1 Publically-funded schools in NZ are ranked by decile for funding purposes. Schools in decile 1 have the largest proportion of students from low socio-economic backgrounds. Schools in decile 10 have the smallest proportion of these students. Each decile contains approximately 10% of schools. http://www.education.govt.nz/school/running-a-school/resourcing/operational-funding/school-decile-ratings/
