# Supplementary material for: Children’s everyday exposure to food marketing: an objective analysis using wearable cameras
Source: Int J Behav Nutr Phys Act. 2017 Oct 8;14:137. doi: 10.1186/s12966-017-0570-3 (PMC5632829; doi:10.1186/s12966-017-0570-3)
Supplement: Supplementary file 1 — Number of children and photos by ethnicity/school decile sampling stratum, and mean number of photos per child in each stratum. (DOCX 15 kb) [file 12966_2017_570_MOESM1_ESM.docx]

Additional file 1. Number of children and photos by ethnicity/school decile sampling stratum, and mean number of photos per child in each stratum.

|  |  |  |  | Number of photos | |
| --- | --- | --- | --- | --- | --- |
| School decile | Ethnic group | N children | N schools | Total | Mean |
|  |  |  |  | for stratum | per child |
| Low (1-3) |  |  |  |  |  |
|  | NZ European | 17 | 2 | 114,262 | 6,721 |
|  | Māori | 25 | 4 | 157,825 | 6,313 |
|  | Pacific | 20 | 5 | 140,236 | 7,011 |
|  |  |  |  |  |  |
| Middle (4-7) |  |  |  |  |  |
|  | NZ European | 24 | 3 | 236,175 | 9,840 |
|  | Māori | 16 | 3 | 70,211 | 4,388 |
|  | Pacific | 15 | 2 | 91,281 | 6,085 |
|  |  |  |  |  |  |
| High (8-10) |  |  |  |  |  |
|  | NZ European | 25 | 4 | 259,733 | 10,389 |
|  | Māori | 19 | 2 | 174,074 | 9,161 |
|  | Pacific | 7 | 2 | 53,685 | 7,669 |
|  |  |  |  |  |  |
| Total |  | 168 | 27 | 1,297,482 | 7,723 |
