# Supplementary material for: Children’s everyday exposure to food marketing: an objective analysis using wearable cameras
Source: Int J Behav Nutr Phys Act. 2017 Oct 8;14:137. doi: 10.1186/s12966-017-0570-3 (PMC5632829; doi:10.1186/s12966-017-0570-3)
Supplement: Supplementary file 2 — Median and interquartile range of per-child rates of exposure per day to core and non-core items, by school. (DOCX 15 kb) [file 12966_2017_570_MOESM2_ESM.docx]

Additional file 2. Median and interquartile range of per-child rates of exposure per day to core and non-core items, by school

|  |  | Core Foods |  | Non-core Foods |
| --- | --- | --- | --- | --- |
| School stratum | Ethnicity | Rate per day* [IQR] |  | Rate per day* [IQR] |
|  |  |  |  |  |
|  |  |  |  |  |
| Low deciles (1-3) | NZ European | 6.1 [1.8 - 13.6] |  | 16.4 [11.2 - 19.4] |
|  | Māori | 8.5 [5.4 - 15.6] |  | 16.3 [11.0 - 26.4] |
|  | Pacific | 9.1 [2.9 - 12.7] |  | 20.6 [11.6 - 35.6] |
|  |  |  |  |  |
| Middle (4-7) | NZ European | 5.9 [3.9 - 12.1] |  | 24.5 [13.6 - 36.6] |
|  | Māori | 6.6 [1.5 - 10.3] |  | 27.0 [19.0 - 46.2] |
|  | Pacific | 6.2 [1.0 - 10.8] |  | 15.9 [6.7 - 24.8] |
|  |  |  |  |  |
| High deciles (8-10) | NZ European | 10.2 [4.6 - 16.9] |  | 23.5 [16.6 - 32.2] |
|  | Māori | 11.9 [6.4 - 19.6] |  | 30.4 [21.3 - 45.9] |
|  | Pacific | 12.4 [5.0 - 14.5] |  | 28.9 [17.4 - 31.5] |
|  |  |  |  |  |
| Total |  | 9.4 [4.2 - 15.0] |  | 23.5 [15.8 - 34.0] |

* Rate of marketing exposures per day (calculated as rate per 10 hours of photographs)
