# Supplementary material for: Children’s everyday exposure to food marketing: an objective analysis using wearable cameras
Source: Int J Behav Nutr Phys Act. 2017 Oct 8;14:137. doi: 10.1186/s12966-017-0570-3 (PMC5632829; doi:10.1186/s12966-017-0570-3)
Supplement: Supplementary file 3 — Mean rate of core and non-core food marketing exposures (per day, with 95% CI, from Poisson regression) by setting with aggregated and detailed setting information (with percentage share of all exposures by setting). (DOCX 16 kb) [file 12966_2017_570_MOESM3_ESM.docx]

Additional file 3. Mean rate of core and non-core food marketing exposures (per day, with 95% CI, from Poisson regression) by setting with aggregated and detailed setting information (with percentage share of all exposures by setting).

|  | Core Foods | |  | Non-core Foods | |
| --- | --- | --- | --- | --- | --- |
| Setting** | Rate per day* (95% CI) | % of total |  | Rate per day* (95% CI) | % of total |
|  |  |  |  |  |  |
| Total | 12.3 (8.7, 17.4) |  |  | 27.3 (24.8, 30.1) |  |
|  |  |  |  |  |  |
| **Home** | **5.5 (4.6, 6.6)** | **44.9%** |  | **8.9 (7.9, 10.1)** | **32.8%** |
| **School** | **5.3 (2.9, 9.5)** | **42.9%** |  | **5.3 (4.2, 6.8)** | **19.5%** |
| **Food venues** | **0.2 (0.1, 0.4)** | **1.7%** |  | **2.7 (1.5, 4.7)** | **9.7%** |
| Fast food indoor | 0.1 (0.0, 0.2) | 0.6% |  | 1.8 (0.9, 3.7) | 6.6% |
| Full service restaurant | 0.0 (0.0, 0.1) | 0.2% |  | 0.5 (0.3, 0.9) | 1.9% |
| Fresh food market | 0.1 (0.0, 0.3) | 0.9% |  | 0.3 (0.1, 1.1) | 1.2% |
| **Recreation venues** | **0.4 (0.3, 0.7)** | **3.5%** |  | **2.1 (1.1, 3.8)** | **7.6%** |
| Sport | 0.1 (0.1, 0.3) | 1.1% |  | 0.4 (0.2, 0.7) | 1.5% |
| Outdoor recreation | 0.1 (0.0, 0.2) | 0.8% |  | 0.5 (0.2, 1.0) | 1.9% |
| Community venue | 0.2 (0.1, 0.5) | 1.6% |  | 1.1 (0.4, 3.3) | 4.2% |
| **Other public spaces** | **0.9 (0.5, 1.5)** | **7.0%** |  | **8.3 (6.0, 11.4)** | **30.4%** |
| Street | 0.2 (0.1, 0.4) | 1.6% |  | 2.0 (1.1, 3.6) | 7.3% |
| Shop front | 0.3 (0.2, 0.5) | 2.5% |  | 3.3 (2.3, 4.8) | 12.2% |
| Shopping mall | 0.0 (0.0, 0.2) | 0.4% |  | 0.5 (0.2, 1.3) | 1.8% |
| Private transport | 0.1 (0.1, 0.4) | 1.2% |  | 0.9 (0.6, 1.3) | 3.2% |
| Public transport facility | 0.1 (0.0, 0.3) | 0.5% |  | 0.3 (0.1, 0.7) | 1.1% |
| Public transport (onboard) | 0.0 (0.0, 0.0) | 0.0% |  | 0.3 (0.0, 1.9) | 1.1% |
| Other Retail | 0.1 (0.0, 0.3) | 0.9% |  | 1.0 (0.6, 1.6) | 3.6% |
|  |  |  |  |  |  |

* Rate of marketing exposures per day (calculated as rate per 10 hours of photographs)

** Aggregated/higher level settings are presented in bold; sub-levels are not bolded.
