# Supplementary material for: Children’s everyday exposure to food marketing: an objective analysis using wearable cameras
Source: Int J Behav Nutr Phys Act. 2017 Oct 8;14:137. doi: 10.1186/s12966-017-0570-3 (PMC5632829; doi:10.1186/s12966-017-0570-3)

Additional file 4. Mean rate (and 95% CI) of core and non-core marketing exposures per day (10 hours of photographs), by school decile stratum and ethnicity of child.


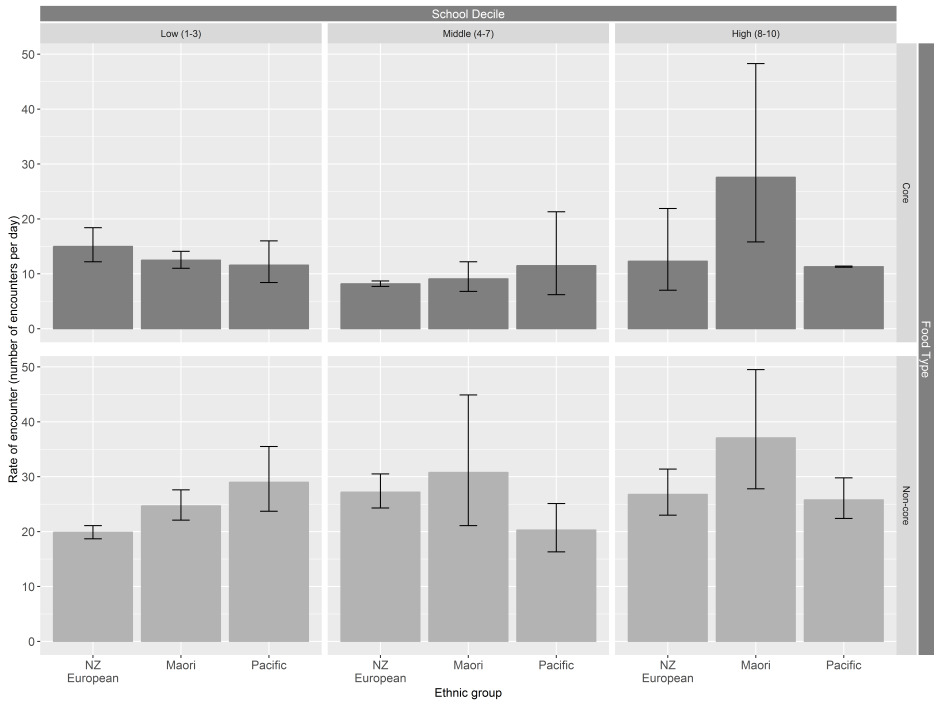

Supplement: Supplementary file 4 — Mean rate (and 95% CI) of core and non-core marketing exposures per day (10 h of photographs), by school decile stratum and ethnicity of child. (DOCX 98 kb) [file 12966_2017_570_MOESM4_ESM.docx]
