# Supplementary material for: Children’s everyday exposure to food marketing: an objective analysis using wearable cameras
Source: Int J Behav Nutr Phys Act. 2017 Oct 8;14:137. doi: 10.1186/s12966-017-0570-3 (PMC5632829; doi:10.1186/s12966-017-0570-3)
Supplement: Supplementary file 5 — Rate ratios for differences in non-core food marketing exposures (from Poisson regression, with 95% CI) by interaction school decile group and ethnicity, adjusted for gender and age. (DOCX 15 kb) [file 12966_2017_570_MOESM5_ESM.docx]

Additional file 5. Rate ratios for differences in non-core food marketing exposures (from Poisson regression, with 95% CI) by interaction school decile group and ethnicity, adjusted for gender and age.

| School decile | Ethnic group |  | Rate ratio (95% CI) |
| --- | --- | --- | --- |
|  |  |  | within school stratum |
| Low (1-3) |  |  |  |
|  | NZ European |  | 1 (Reference) |
|  | Māori |  | 1.20 (0.97, 1.47) |
|  | Pacific |  | 1.50 (1.19, 1.89) |
|  |  |  |  |
| Middle (4-7) |  |  |  |
|  | NZ European |  | 1 (Reference) |
|  | Māori |  | 1.19 (0.77, 1.84) |
|  | Pacific |  | 0.76 (0.59, 0.96) |
|  |  |  |  |
| High (8-10) |  |  |  |
|  | NZ European |  | 1 (Reference) |
|  | Māori |  | 1.38 (1.00, 1.90) |
|  | Pacific |  | 0.91 (0.66, 1.26) |
|  |  |  |  |
| Variable (rest of model) | |  | Non-core Foods |
|  |  |  | Rate ratio (95% CI) |
|  |  |  |  |
| Gender |  |  |  |
| Female |  |  | 1 (Reference) |
| Male |  |  | 1.05 (0.82, 1.33) |
|  |  |  |  |
| Age (per year) |  |  | 0.94 (0.75, 1.16) |
|  |  |  |  |
